# What makes an eLife paper in epidemiology and global health?

**DOI:** 10.7554/eLife.11326

**Published:** 2015-10-07

**Authors:** Mark Jit, Eduardo Franco, Prabhat Jha

**Affiliations:** Department of Infectious Disease Epidemiology, London School of Hygiene and Tropical Medicine, London, United Kingdom and the Modelling and Economics Unit, Public Health England, London, United KingdomMark.Jit@lshtm.ac.uk; Division of Cancer Epidemiology, McGill University, Montreal, Canadaeduardo.franco@mcgill.ca; Center for Global Health Research, Saint Michael's Hospital, Toronto, Canada and Dalla Lana School of Public Health, University of Toronto, Toronto, Canadajhap@smh.ca

**Keywords:** research, scientific publishing, peer review

## Abstract

The best papers provide evidence that can be used to make changes that improve the health and lives of people around the world.

Earlier this year an editorial explained what eLife editors look for in a paper: ‘For us, the ideal eLife paper presents an accurate description of data that makes others in the field think differently and moves the field forward’ (Malhotra and Marder, 2015). Here we outline how this applies to papers in epidemiology and global health.

First, as with all manuscripts submitted to eLife, we ask if the submission addresses an important question and uses study designs that provide a reasonably clear answer to that question. The disciplines of epidemiology and global health sit squarely on the boundary between the natural, clinical and social sciences, so a range of study designs can be used. Experimental, observational and theoretical lines of enquiry may all be appropriate; both qualitative and quantitative methods may also be used. Health and disease are determined by a host of physical, biological, psychological, technological, social, economic and political factors, and these factors need to be investigated both individually and in combination. So eLife has no pre-conceived notions of what constitutes a good epidemiology or global health paper; certainly we do not limit ourselves to experimental studies or studies rooted in the natural sciences alone. Indeed, we welcome the best papers across the entire gamut of disciplines that contribute to these fields, including those that use rigorous scientific methods to explore the impact of behavioural and socioeconomic factors on health.

Second, research in epidemiology and global health often directly informs decisions at the hospital bedside or at the planning office. Like the editors who handle submissions in other areas of the life and biomedical sciences, we seek submissions that represent the best quality science in terms of rigor and insight. However, researchers in epidemiology and global health have an additional responsibility to maximise the potential of their work to save lives and improve health. Hence we privilege submissions that have the greatest potential impact on health around the world, especially the health of the worst off. This might be an analysis that could lead to a new approach for cancer care or malaria prevention that could save millions of lives, or it could be the discovery of a risk factor for an orphan disease which we previously had little hope of preventing or curing. This does not exclude methodological papers that may not immediately save lives but are highly likely to enable later studies that do. We also welcome papers that are so clear and persuasive in the way they express important truths that they will be read and re-read by clinicians and policy-makers. And since eLife is an open access journal, all articles are freely available to everyone.

Third, we recognise that excellent science can look different in epidemiology and global health because studies are often less precise and controllable than in many of the biological sciences, let alone the physical sciences. Preliminary findings often need to be corroborated with larger, better controlled studies and, eventually, the syntheses of many pieces of relevant evidence. Hence we welcome reports of high-quality clinical trials, along with major reviews and meta-analyses that provide the strength of evidence that will finally allow the findings of smaller studies to be translated into life-saving decisions. Ultimately, we ask ourselves: does this manuscript constitute a substantial step towards a clear answer to an important global health question?

Ultimately, we ask ourselves: does this manuscript constitute a substantial step towards a clear answer to an important global health question?

Much in epidemiology is of corroborative value. Given the bluntness of our toolbox, epidemiological findings must be replicated before they can be considered as evidence for the need to change practice in medicine and public health. We respect that but believe that papers that attempt to corroborate previous findings without taking a substantial step forward, or bringing a new angle to the problem, will have a better home in specialty journals. We seek to reward innovative and smart explorations of population health data. Sometimes the intellectual excitement that a paper elicits does not come from the sophistication of the methodology but from the clever use of simple methods to reveal a possibly causal association that was hidden from view in previous investigations. Eureka moments exist in epidemiology; we wish to display them prominently in eLife.

We recognise and celebrate the fact that global health is now a truly international endeavour, and we are especially keen to receive submissions from the low- and middle-income nations that are under-represented in most journals, including eLife. In the same vein, we think it stands to reason that papers using new data collected in these countries should normally include co-authors from the countries whose health-related data are the focus of the investigation. How else could these studies have captured the appropriate context for an in-depth exploration of the research problem?

In conclusion, when making decisions about submissions in epidemiology and global health, we look for all the things you would expect to see in papers in a good journal—such as a clear question, clever insights and clear clarity of logic—combined with results and findings that have the potential to improve human health.
